# Omega-3 fatty acid-derived resolvins and protectins in inflammation resolution and leukocyte functions: targeting novel lipid mediator pathways in mitigation of acute kidney injury

**DOI:** 10.3389/fimmu.2013.00013

**Published:** 2013-01-30

**Authors:** Song Hong, Yan Lu

**Affiliations:** Neuroscience Center of Excellence, Health Science Center, Louisiana State UniversityNew Orleans, LA, USA

**Keywords:** resolvins, protectins/neuroprotectins, maresins, 14*S*,21*R*-diHDHA, inflammation-resolution, kidney-injury, fibrosis, leukocytes

## Abstract

Inflammation, in conjunction with leukocytes, plays a key role in most acute kidney injury (AKI). Non-resolving renal inflammation leads to chronic fibrosis and renal failure. Resolvin D series (RvDs) and E series (RvEs), protectins, and maresins (MaRs) are endogenous omega-3 fatty acid-derived lipid mediators (LMs) that potently promote inflammation resolution by shortening neutrophil life span and promoting macrophage (Mf) non-phelogistic phagocytosis of apoptotic cells and the subsequent exit of Mfs from inflammatory tissue. 14*S*,21*R*-dihydroxy docosahexaenoic acid (14*S*,21*R*-diHDHA), a Mf-produced autacrine, reprograms Mfs to rescue vascular endothelia. RvD1, RvE1, or 14*S*,21*R*-diHDHA also switches Mfs to the phenotype that produces pro-resolving interleukin-10. RvDs or protectin/neuroprotectin D1 (PD1/NPD1) inhibits neutrophil infiltration into injured kidneys, blocks toll-like receptor -mediated inflammatory activation of Mfs and mitigates renal functions. RvDs also repress renal interstitial fibrosis, and PD1 promotes renoprotective heme-oxygenase-1 expression. These findings provide novel approaches for targeting inflammation resolution and LMs or modulation of LM-associated pathways for developing better clinical treatments for AKI.

## Acute kidney injury: an inflammatory disease

### Acute kidney injury: an unmet medical challenge

Acute kidney injury (AKI), formerly known as “acute renal failure,” causes a decline of kidney function (Bonventre and Yang, 2011). AKI occurs in many conditions, and AKI mortality is quite significant (Bonventre and Yang, 2011). Patients with AKI have a high chance of developing chronic or end-stage renal disease if they survive. Pharmacologic treatment and renal replacement therapy are only preventive or supportive and have not reduced AKI mortality (Negi and Shigematsu, 2012). The current treatment for AKI is still only preventive or supportive (Bonventre and Yang, 2011). Kidney ischemia/reperfusion injury (KIR) is a common cause of AKI (Bonventre and Yang, 2011).

### Inflammation, leukocytes, and inflammation resolution: crucial to acute kidney injury and chronic fibrosis

**Figure 1:**
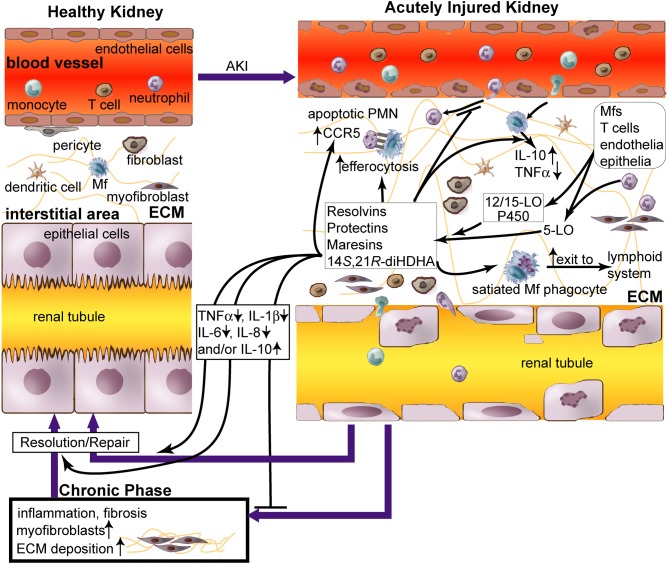
**Role of lipid mediators, resolvins and protectins in inflammation resolution in acute renal injury (AKI).** AKI causes (1) damage of renal vascular endothelium; (2) diapedesis of neutrophils and monocytes through endothelium; (3) accumulation of neutrophils, macrophages (Mfs), dendritic cells (DCs), T cells, fibroblasts, and myofibroblasts in renal tissue; and (4) apoptosis and necrosis of tubular epithelium. Impaired inflammation resolution that occurs during AKI leads to chronic fibrosis [i.e., excessive extracellular matrix (ECM, as light-green lines) produced by myofibroblasts and other cells in the kidneys] and ultimately chronic renal failure (Bonventre and Yang, 2011; Serhan and Petasis, 2011). Although leukocytes cause tissue damage when activated to inflammatory phenotypes by AKI in the initial phase, they could promote inflammation resolution and tissue repair when modulated by pro-resolving lipid mediators, including resolvins and protectins. Resolvins, protectins, maresins, and 14*S*,21*R*-diHDHA are produced by 12/15-lipoxygenase (LO), p450, and/or 5-LO, in trans-cellular or intracellular biosynthetic systems of leukocytes or leukocytes plus endothelia/epiothelia. These lipid mediators act as paracrines and autacrines of leukocytes to promote resolution of AKI-initiated inflammation and fibrosis and rescue of kidney functions. They also inhibit accumulation of neutrophils and Mfs in kidneys during acute inflammation in AKI (Duffield et al., 2006; Tian et al., 2012), promote Mf non-phlogistic efferocytosis of apoptotic neutrophils (Hong et al., 2008), enhance apoptotic leukocyte expression of CCR5 that scavenges critical inflammatory chemokines, and accelerate phagocyte exit from the inflammatory site via the lymphatics (Ariel et al., 2005; Ariel and Serhan, 2007). These lipid mediators increase the level of pro-resolving, anti-fibrotic IL-10 and reduce the levels of inflammatory cytokines TNFα, IL-1β, IL-6, and/or IL-8 in Mfs or renal tissue. Thus these lipid mediators act as effectors for inflammation resolution and injury repair, which restores AKI-damaged kidneys to homeostasis. Administration of these lipid mediators promotes resolution of inflammation and injury in AKI.

Inflammation plays a critical role in pathogenesis and recovery of AKI (Bonventre and Yang, 2011). AKI is characterized by infiltration and activation of leukocytes neutrophils, macrophages (Mfs), dendritic cells (DCs), and lymphocytes as well as damage (apoptosis and necrosis) of vascular endothelia and tubular epithelia (Figure 1). The activated leukocytes produce reactive oxidative species (ROS) and inflammatory factors, both of which damage the surrounding tissue. Mfs and DCs participate in both the innate and adaptive immune responses. Mfs infiltrated into kidneys during the first 48 h after KIR are mainly inflammatory M1 type that injures the tissue, whereas non-inflammatory M2 Mfs predominate later and are correlated with kidney repair (Lee et al., 2011). Regulatory T-cells are protective in AKI (Ko et al., 2010). B-cell deficiency confers protection from KIR injury (Burne-Taney et al., 2003). This type of injury also stimulates expression of adhesion molecules by vascular endothelia, such as ICAM-1 and VCAM-1, promoting leukocyte accumulation around injured sites. The injury-enhanced interaction of endothelia and leukocytes produces inflammatory cytokines, prostaglandins, leukotrienes, and complements, compromising endothelial junctions due to swelling and loss of glycocalyx and actin cytoskeleton. AKI inflammation goes into a positive feedback amplification as more blood leukocytes infiltrate through the vascular endothelial barrier into other renal tissue and become activated until inflammation resolution dominates over the inflammation (Figure 1) (Borgeson and Godson, 2010; Bonventre and Yang, 2011). Tubular epithelia, mesangium, and pericytes also produce inflammatory factors after injury or interaction with leukocytes, such as TNF-α, IL-8, IL-6, and IL-1β, leading escalated kidney inflammation and damage (Figure 1) (Bonventre and Yang, 2011).

While leukocyte-led inflammation causes tissue injury; a natural force for inflammation resolution is gaining ground both in parallel and in series (Serhan et al., 2002; Kieran and Rabb, 2004; Kluth, 2007; Serhan and Petasis, 2011). Certain macrophage phenotypes promote inflammation resolution in AKI (Alikhan et al., 2011; Lee et al., 2011). Particularly, pro-resolving lipid mediators (LMs) are produced by leukocytes or interaction of leukocytes, endothelium, and epithelium (Hong et al., 2003; Serhan and Petasis, 2011). These mediators trigger signaling that reduces production of inflammatory factors, enhances non-phlogistic efferocytosis, and promotes the switch of inflammatory leukocytes to pro-resolution reparative phenotypes (Figure 1, Table 1) (Hong et al., 2008).

**Table 1:** **Selected characteristics of n3-PUFAs-derived lipid mediators**.

**Lipid mediator**	**Pre-cursor**	**Enzyme(s) for biosynthesis**	**Receptor(s)**	**Activate signaling**	**Deactivate signaling**	**Inhibiting inflammatory molecule expression**	**Promoting pro-resolving cytokine expression**
RvD1	DHA	5-LO + (12/15-LO or 15-LO)	FPR2/ALXR			IL-8 (p)	IL-10 (q)
			GPR32			MIP-1β (p)	
		(a–c)	(l)			RANTES (p)	
						IL-6 (p)	
						VCAM-1 (p)	
						TNFα (q)	
						IL-1β (q)	
RvE1	EPA	5-LO + (12/15-LO or 15-LO)	CMKLR1/ChemR23	PI3K	NFκB (e)	IL-8 (p)	IL-10 (q)
				Akt		VCAM-1 (p)	
		(c–e)	BLT1 (e)	ERK1/2 (m)		MIP-1β (p)	
						RANTES (p)	
						TNFα (p)	
						VCAM-1 (p)	
						IL-1β (q)	
PD1/NPD1	DHA	12/15-LO or 15-LO		PI3K	NFκB (o)	COX2 (r)	
				Akt			
		(b, c, f)		mTOR/p70S6K (n)			
14*S*,21*R*-diHDHA	DHA	(12/15-LO or 12-LO) + P450		PI3K			IL-10 (i)
				Akt			
				p38-MAPK			
		(g–k)		(h–k)			

*Notes: (a) (Serhan et al., 2002); (b) (Hong et al., 2003); (c) (Serhan and Petasis, 2011); (d) (Serhan et al., 2000); (e) (Arita et al., 2005a); (f) (Serhan et al., 2006); (g) (Lu et al., 2010); (h) (Tian et al., 2011a); (i) (Tian et al., 2011b); (j) (Tian et al., 2010); (k) (Tian et al., 2012); (l) (Spite et al., 2009); (m) (Ohira et al., 2010); (n) (Faghiri and Bazan, 2010); (o) (Marcheselli et al., 2003); (p) (Tian et al., 2009); (q) (Schif-Zuck et al., 2011); (r) (Mukherjee et al., 2004). BLT1, leukotriene B4 receptor; CMKLR1/ChemR23, chemokine-like receptor 1/chemerin receptor 23; ERK1/2, extracellular signal-regulated protein kinases 1 and 2; FPR2, formyl peptide receptor 2; GPR32, G protein-coupled receptor 32; MAPK, mitogen-activated protein kinases; MIP-1β, macrophage inflammatory protein 1β; mTOR/p70S6K, mammalian target of rapamycin/p70 ribosomal S6 kinase; PI3K, phosphatidylinositol 3-kinase; RANTES, regulated upon activation normal T cell expressed and presumably secreted*.

Renal chronic fibrosis is the formation of excessive fibrous connective tissue in kidneys due to excessive accumulation in the extracellular matrix in response to chronic inflammation or repeated injury. Although appropriate local and transient renal fibrosis is needed for repair in the early phase of AKI, chronic fibrosis is a major detrimental feature in the later phases (Borgeson and Godson, 2010). Chronic fibrosis can eventually lead to end-stage renal failure. Leukocytes play a crucial role in renal fibrosis during AKI (Borgeson and Godson, 2010) (Figure 1). Lipoxin (LX) A_4_, protectin/neuroprotectin D1 (PD1/NPD1), or resolvin D1 (RvD1) suppresses chronic fibrosis in KIR-injured kidneys (Godson et al., 2000; Duffield et al., 2006; Borgeson and Godson, 2010; Borgeson et al., 2011). Mfs interact with fibroblasts and pericytes, the key cells that can transdifferentiate into fibrosis-forming myofibroblasts (Figure 1) (Duffield, 2010). Mfs under inflammatory activation produce pro-fibrotic factors—such as TGF-β1, IL-13, and platelet-derived growth factor (PDGF) (Ko et al., 2008)—and also contribute to renal fibrosis (Young et al., 1995). Moreover, Mf depletion reduces renal fibrosis, but Mfs also can produce anti-fibrotic factors, such as IL-10, specific matrix metalloproteinases, and Endo180, in addition to phogocytose fibrotic extracellular matrix, apoptotic myeofibroblasts, and tissue debris (Vernon et al., 2010). Adoptive transfer of Mfs into mice at the chronic inflammation phase ameliorates chronic renal fibrosis (Nishida et al., 2005). This demonstrates that certain Mf phenotypes contribute to the prevention or resolution of chronic renal fibrosis.

In the following sections, we will present a concise review on omega-3 polyunsaturated fatty acids (n3-PUFA)-derived LMs that promote the resolution of inflammation and chronic fibrosis as well as repair in AKI.

## Specialized anti-inflammatory, pro-resolving lipid mediators derived from n3-PUFAs: resolvins, protectins, and maresins (Figure 1, Table 1)

### Chemical structures and formation *In vivo* and *In vitro*

Docosahexaenoic acid (DHA) and eicosapentaenoic acid (EPA), the major n3-PUFAs present in fish oils, have beneficial effects that could prove helpful in preventing and/or treating inflammatory diseases (Kelley et al., 1999; Simopoulos, 2002). As such, the molecular and cellular mechanisms behind these beneficial effects are of significant interest and have been explored (Bazan et al., 1984; Serhan et al., 2000, 2002; Hong et al., 2003; Marcheselli et al., 2003; Serhan, 2011). The structures and bioactivities of several families of novel n3-PUFAs-derived LMs that are both anti-inflammatory and pro-resolving have been discovered (Serhan, 2011). Some of these compounds were termed resolvins since they are formed in the resolution phase of inflammation and potently promote resolution (Serhan et al., 2002). Another DHA-derived LM, 10,17S-docosatriene, was discovered (Serhan, 2011). It was termed neuroprotectin D1 if generated in neural tissue for its protection in neurons, glial cells, and brain stroke; or protectin D1 for other tissue (Bazan, 2005; Serhan, 2011).

Resolvin D series (RvDs) are derived from DHA. During inflammation, endogenous DHA is converted to 17*S* hydroxyl-containing RvDs (RvD1–RvD6) and docosa-conjugated triene-containing PD1/NPD1 via 15-lipoxygenase (LO) (15*S*-lipoxygenation)-initiated biochemical pathways (Serhan et al., 2002; Hong et al., 2003; Marcheselli et al., 2003) or to 14*S* hydroxyl-containing maresins (MaRs) via 12-LO (12*S-lipoxygenation)*-initiated biochemical pathways. 5-LO catalyzes sequentially with 15-LO or 12/15-LO, generating RvDs (Hong et al., 2003) and some MaRs (Serhan et al., 2009). PD1, in isolated human cells and murine cells, was found to be 10*R*,17*S*-dihydroxy-docosa-4*Z*, 7*Z*, 11*E*, 13*E*, 15*Z*, 19*Z*-hexaenoic acid (Serhan et al., 2006). RvD1, RvD2, PD1/NPD1, and/or their biosynthetical pathway marker 17*S*-hydroxyl DHA (17*S*-HDHA) have been found in blood (Hong et al., 2003), ischemia-injured brains, retinal pigment epithelial cells, and/or AKI kidneys (Marcheselli et al., 2003; Mukherjee et al., 2004; Duffield et al., 2006; Bazan et al., 2011), demonstrating the existence of these compounds and/or pathways in injured tissue or cells. The interaction of endothelial cells and leukocytes promotes their biosynthesis (Tian et al., 2009) (Figure 1). RvE1 and 17*R*-hydroxyl epimers of RvDs and PD1, on the other hand, are generated through 15*R*-lipoxygenation pathways catalyzed by aspirin-acetylated cyclocygenase-2 (COX-2) or cytochrome P450 (in contrast to the typical 15-LO-catalyzed 15*S*-lipoxygenation of arachidonic acid) (Serhan et al., 2000, 2006). They were found in exudates, blood, and brains of humans and animals treated with aspirin (Serhan, 2011; Bazan et al., 2012). This provides new molecular insights for aspirin-based anti-inflammatory medication besides inhibiting COXs to produce inflammatory prostaglandins and thromboxins.

Recently we found several additional new pro-healing LMs: 14*S*,21*R*-dihydroxy-docosa-4*Z*, 7*Z*, 10*Z*, 12*E*, 16*Z*, 19*Z*-hexaenoic acid (14*S*,21*R*-diHDHA) and its epimers (Lu et al., 2010; Tian et al., 2011a,b). 14*S*,21*R*-diHDHA, as a positional isomer of maresin-1 (Serhan et al., 2009), is generated from DHA in Mfs, neutrophils, and cutaneous wounds. 12-LO and P450 catalyze sequentially to convert DHA to 14*S*,21*R*-diHDHA and 14*S*,21*S*-diHDHA through the intermediacy of 14*S*-HDHA (formed via 12*S*-lipoxygenation from DHA) (Lu et al., 2010; Tian et al., 2011a).

### Bioactions

Resolvins, protectins, and MaRs recapitulate beneficial bioactions of DHA or EPA with several order-of-magnitudes higher potency (in nanomolar and picomolar range) compared to their precursors (DHA or EPA) (Serhan and Petasis, 2011). These LMs have potent anti-inflammatory and pro-resolving effects, since they inhibit inflammatory factor expression and neutrophil infiltration, and since they promote non-phlogistic Mf phagocytosis of apoptotic cells (Serhan and Petasis, 2011). Such actions have been revealed in many *in vivo* models of inflammatory diseases, as well as *in vitro* experiments on diverse types of cells critical to these diseases. These actions include dermal inflammation, peritonitis, periodontitis, colitis and intestinal inflammation, asthma and airway inflammation, cystic fibrosis, acute lung or kidney injury, glomerulonephritis, and brain stroke (Marcheselli et al., 2003; Serhan and Petasis, 2011). RvE1 and its analogs are currently undergoing clinic trials for diseases of the eye, lung, kidney, skin, and intestines (Serhan and Petasis, 2011). Bazan et al. discovered that PD1/NPD1 resolves inflammation in brain and eye (Marcheselli et al., 2003; Mukherjee et al., 2004; Lukiw et al., 2005). PD1 or LXA_4_ blocks inflammatory cytokine secretion from human T-cells and enhances CCR5 expression on apoptotic PMN (Figure 1), which accelerates clearance of inflammatory CCR5 ligands (Ariel et al., 2003, 2005). PD1 also promotes T-cell apoptosis (Ariel et al., 2005), as well as reduces the neutrophil lifespan in peritonitis (Bannenberg et al., 2005) and neutrophil-survival signaling for IL-1β (Hong et al., 2003). RvE1 promotes phagocytosis-induced neutrophil apoptosis and resolution of pulmonary inflammation (El Kebir et al., 2012). Several comprehensive reviews on these mediators are already available (Borgeson and Godson, 2010; Serhan and Petasis, 2011; Bazan, 2012).

14*S*,21-diHDHA and 14*R*,21-diHDHA promote or restore wound healing (Lu et al., 2010) impaired by alcohol intoxication (Tian et al., 2010) or diabetes (Tian et al., 2011a). Also, 14*S*,21*R*-diHDHA enhances VEGF release, vascularization, and migration of endothelial cells in diabetic mice. It also remedies angiogenic and pro-healing functions of mesenchymal stem cells (MSCs) and Mfs attenuated by diabetes, including their production of VEGF and/or IL-10 (Tian et al., 2011b). 14*S*,21*R*-diHDHA reduces hyperglycemia-induced ROS generation by inflammatorily-activated Mfs (Tian et al., 2011b). Thus, 14*S*,21*R*-diHDHA is a specific pro-resolving LM that may promote the protection or repair of the renal endothelium and epithelium during AKI.

### Receptors (Table 1)

Two G-protein-coupled receptors have been identified for RvE1: (**1**) BLT1 in neutrophils; and (**2**) CMKLR1/ChemR23 in Mfs and DCs (Arita et al., 2005a,b). RvD1 has also been reported to interact with both FPR2 or LXA_4_ receptor (ALXR) and GPR32 in phagocytes (Spite et al., 2009). ALXR is expressed in neutrophils (Fiore et al., 1994) and monocytes (Maddox et al., 1997), and it activates T-cells (Ariel et al., 2003), intestinal or bronchial epithelial cells (Bonnans et al., 2003; Kucharzik et al., 2003), and renal mesangial cells (McMahon et al., 2000; Maderna and Godson, 2009), implying ALXR existence in renal podocytes and tubular epithelium. PD1/NPD1 stereoselectively and specifically binds with retinal pigment epithelial cells and neutrophils, suggesting specific receptors for NPD1 in both the immune and visual systems (Marcheselli et al., 2010). However, the exact NPD1 receptor(s) needs to be identified. The receptors of other resolvins, protectins, and marsins are likely to exist based on their structure-activity association, but have not been discovered yet.

### Cell signaling (Table 1)

Through CMKLR1 or BLT1 receptors, RvE1 represses the activation of NFκB (Arita et al., 2005a, 2007), a crucial regulator of innate immune responses in kidneys (Mulay et al., 2012). NPD1 or RvE1-CMKLR1 interactions activate PI3K and Akt, which involves mTOR signaling; RvE1 also activates ERK1/2 (Faghiri and Bazan, 2010) 14*S*,21*R*-diHDHA activates PI3K, Akt, and P38-MAPK, but not ERK1/2 (Tian et al., 2010, 2011a, 2012). PI3K-Akt signaling regulates cell survival, and activation of MAPK pathways is essential in wound healing and associated angiogenesis (Tian et al., 2010, 2011a). These signaling systems are relevant to AKI (Borgeson and Godson, 2010; Tian et al., 2012).

### Metabolic deactivation

RvD1 is converted by eicosanoid oxidoreductases (EORs) to 17-oxo-RvD1 and 8-oxo-RvD1. The former is an inactivation metabolite, while the latter is still effective in suppressing neutrophil infiltration (Sun et al., 2007). RvE1 is metabolized to 12-oxo-RvE, 18-oxo-RvE1, 10,11-dihydroxy RvE, 19-hydroxy RvE1, 20-hydroxy RvE1 in tissue or cells, of which the first four metabolites are inactive partially or completely in inflammation resolution, and thus are representative for RvE1 metabolic deactivation (Arita et al., 2006; Hong et al., 2008). Human neutrophils convert PD1 to its omega-22 hydroxy product (Serhan and Petasis, 2011). The metabolic deactivation of resolvins could be excessively up-regulated in pathological conditions, resulting in their deficiency, or diminishing the pharmacological efficacy of administered resolvins. Molecular engineering has been used to overcome this problem; for example, A *p*-fluorophenoxyl added to RvE1 ω-terminal blocks the critical metabolic inactivation of RvE1 without attenuating the anti-inflammatory pro-resolving activities (Arita et al., 2005a; Hong et al., 2008).

## Resolvin D series and protectin D1 resolve inflammation and mitigate AKI (Figure 1, Table 1)

Based on the findings that DHA-derived RvDs and PD1 promote inflammation resolution (Serhan, 2011) and DHA supplementation reduces KIR injury in dogs and rats (Neumayer et al., 1992; Kielar et al., 2003), Duffield and colleagues studied the treatment of murine KIR with RvDs and PD1. The study showed that administration of RvDs (RvD1:RvD2:RvD3 = 1:2:1), RvD1, or PD1 attenuates functional and morphological kidney injury, reduces accumulation of inflammatory neutrophils and Mfs, and suppresses TLR-mediated activation of Mfs. TLR signaling in Mfs and lymphocytes is involved in sustained chronic inflammation (Foell et al., 2007; Kato et al., 2008). RvDs treatment until 72 h after ischemia inhibits renal interstitial chronic fibrosis (Duffield et al., 2006). Interstitial chronic fibrosis and persistent leukocyte infiltration (chronic inflammation), resulting from AKI, leads to scarring and chronic renal failure (Morgera et al., 2002; Duffield and Bonventre, 2004). RvDs and PD1 likely have additional cellular sites of action in the kidney, e.g., on the endothelium and vascular tone, interstitial fibroblasts, mesangial cells, pericytes, DCs, and T-cells because of their pro-resolving and anti-fibrotic ability (Duffield et al., 2006). They may modulate the actions of monocytes/Mfs and neutrophils in the kidney. Hassan and Gronert found that PD1 amplified renoprotective heme-oxygenase-1 (HO-1) expression in ischemia-injured and non-injured kidneys, while PD1 inhibited neutrophil infiltration in murine KIR (Hassan and Gronert, 2009). These results support their notion that the interaction of the 12/15-LO and HO-1 systems provides a positive feedback loop that amplifies anti-inflammatory, pro-resolving signals.

Godson and colleagues found that arachidonic acid-derived LXs are pro-resolving in several types of renal injury; LXs play a reparative role in glomerulonephritis, and reduce proteinuria, glomerular inflammation, and mesangial cell proliferation (Kieran et al., 2004; Wu et al., 2006; Borgeson and Godson, 2010). LXs are protective against murine KIR, where a LX-stable analogue gives functional and morphological protection and attenuates inflammatory cytokine responses (Leonard et al., 2002). LXs also up-regulate genes of tight-junction proteins claudin 1, 3, and 7, which likely reduce inflammatory leukocyte infiltration; Moreover, they found that LXs attenuate renal chronic fibrosis and related gene expression in mesangial cells (Borgeson and Godson, 2010). LXA_4_ analog or RvE1 remarkably prolong renal allograft survival in mice, which is consistent with LXA_4_ inhibition of calcineurin activity and inflammatory cytokine release by human neutrophils. Also, RvE1 counter-regulates leukocytes partially via increased LXA_4_ biosynthesis (Levy et al., 2011). Since AKI is the major complication of renal allograft transplantation (Bellomo et al., 2012), these results further demonstrate the effectiveness of LXA_4_ or RvE1 in reducing AKI. LX actions converge with the pro-resolving characteristics of RvD1, as LXA_4_ and RvD1 both activate the same G-protein coupled receptors ALXR/FPR2 and GPR32.

## 14*S*,21*R*-diHDHA promotes mesenchymal stem cells in resolution of inflammation and prevention of AKI

MSCs have shown potential to resolve inflammation and repair injury in renal failure (Togel et al., 2005). MSCs treated with 14*S*,21*R*-diHDHA more efficiently inhibit KIR-induced elevation of serum creatinine levels and reduce renal tubular cell death, as well as infiltration of neutrophils, Mfs, and DCs to renal tissue. Conditioned media from 14*S*,21*R*-diHDHA-treated MSCs reduce the generation of TNF-α and ROS by Mfs under KIR conditions. Infusion of 14*S*,21*R*-diHDHA-treated MSCs more efficiently reduce KIR-renal damage compared to untreated MSCs. Treated MSCs are resistant to apoptosis *in vivo* (when transplanted under capsules of AKI-injured kidneys) and *in vitro* (when cultured under simulated KIR conditions). This enhancement of MSC viability involves PI3K-Akt signaling. Additionally, treatment of MSCs with 14*S*,21*R*-diHDHA promotes secretion of renotrophic hepatocyte growth factor and insulin growth factor-1. In brief, 14*S*,21*R*-diHDHA promotes MSC amelioration of AKI (Tian et al., 2012).

## Resolvins, protectins, and maresins act on leukocytes related to fibrosis in AKI

Although the mechanisms that resolvins and PD1 use to reduce renal chronic fibrosis in AKI (Duffield et al., 2006) remain to be further delineated, the following findings provide hints for future research on this subject. PD1, RvD1, or RvE1 switches Mfs to pro-resolving phenotypes, including CD11b^low^ Mfs, which are more capable in efferocytosis and emigration to lymphoid organs for inflammation resolution (Figure 1) (Schwab et al., 2007; Schif-Zuck et al., 2011; Ariel and Serhan, 2012). RvD1, RvE1, or 14*S*,21*R*-diHDHA induces Mfs to produce more anti-fibrotic IL-10 (Schif-Zuck et al., 2011; Tian et al., 2011b). These pro-resolving LMs, acting in concert in AKI, not only inhibit inflammation, but also shift the macrophage roles from pro-inflammatory (M1) or pro-fibrotic phenotypes to phenotypes that promote resolution as well as anti-fibrotic, regulatory functions (Figure 1, Table 1) (Duffield et al., 2006; Serhan and Petasis, 2011; Ariel and Serhan, 2012).

## Concluding remarks and perspectives

The discoveries of n3-PUFA-derived resolvins, protectins, and MaRs in the last two decades have provided unconventional knowledge and opened new frontiers for understanding the mechanisms involved in inflammation resolution. These LMs are produced endogenously by enzymes in leukocytes and tissue and act as paracrines and autacrines of leukocytes. Experiments have already shown that selected LMs promote resolution of AKI-caused inflammation and chronic fibrosis and rescue kidney function. LMs inhibit recruitment of neutrophils and monocytes to kidneys during acute inflammation, and they likely switch Mfs and T-cells toward anti-inflammatory pro-resolving phenotypes in AKI, as observed in other inflammatory conditions (Figure 1, Table 1). Mechanisms behind the actions of these LMs and their regulatory roles on leukocytes provide the basis for developing leukocyte-related modalities for efficient AKI treatment. These LMs or their mimics may be of therapeutic importance for treating AKI. More studies need to be conducted to further delineate the kinetic process for these LMs in reprogramming the phenotypes of leukocytes, which regulate the resolution of renal inflammation and chronic fibrosis and recover renal functions in AKI. Additional up-stream or down-stream signaling pathways involved should also be studied, as they may yield novel mechanistic targets and insights for AKI treatment.

### Conflict of interest statement

The authors declare that the research was conducted in the absence of any commercial or financial relationships that could be construed as a potential conflict of interest.
